# *Yokenella regensburgei* Septicemia in a Chinese Farmer Immunosuppressed by HIV: A Case Report and Literature Review

**DOI:** 10.1155/2017/5962463

**Published:** 2017-05-16

**Authors:** Xiangbo Chi, Min Liu, Yaokai Chen

**Affiliations:** Chongqing Public Health Medical Center, Chongqing, China

## Abstract

*Yokenella regensburgei* is a member in the family* Enterobacteriaceae* and a few cases have been reported in immunocompromised hosts. Herein, we described a case of septicemia in a human immunodeficiency virus (HIV) infected patient in South West China, which is the first reported case of* Y. regensburgei* infection in HIV-infected populations. We then reviewed the literature on all the reported cases of* Y. regensburgei* infection worldwide and presented some common features of them. Our case report and literature review will help increase the knowledge of the bacterium* Y. regensburgei* and its clinical implications.

## 1. Introduction

*Yokenella regensburgei* belongs to the family* Enterobacteriaceae* and there is no strong evidence to support its clinical importance. There have been no* Y. regensburgei* infection reports in human immunodeficiency virus (HIV) infected patients, although a few case reports have suggested it is an opportunistic pathogen. Herein, we describe a case of septicemia in South West China caused by* Y. regensburgei* in a patient with HIV infection and present a review on* Y. regensburgei *literature.

## 2. Case Report

A 38-year-old male with a 20-year history of injection drug use was admitted to Chongqing Public Health Medical Center (Chongqing, China) in October 2013 due to a fever in absence of chills and cough. Two months prior to his admission, the patient was admitted to another hospital for epistaxis, dyspnea and odynuria with anemia, leucopenia, thrombocytopenia, and urinary tract infection, which improved with blood transfusions and use of antibiotics (levofloxacin injection, 0.2 grams each time, twice a day, for 14 days). His white blood cell counts ranged from 2.0 × 10^9^ to 2.5 × 10^9^ cells/liter during the period of 1 year prior to this admission.

He was confirmed to be HIV-positive since 2009 and received one year later an antiretroviral regimen containing stavudine, lamivudine, and efavirenz, which was discontinued in March 2013 due to renal impairment. He had been on methadone maintenance treatment for about 1 year.

He worked as a farmer. Family and social history were noncontributory. He smoked 10 cigarettes a day on average but rarely consumed alcohol.

On physical examination, the patient appeared pale and uncomfortable. Temperature was 38.7°C, blood pressure was 127/84 mmHg, heart rate was 104/min, and respiratory rate was 20/min. Oxygen saturation was 100% on 3 liters/minute nasal cannula. Cardiac exam was within normal limits and pulmonary and abdominal exams were benign. His skin was intact and no ulceration was found in his mouth. Chest computed tomography was performed and no abnormalities were found.

Blood examinations revealed anemia with hemoglobin of 42 g/liter (reference range, 130 to 175 grams/liter). Blood tests also revealed thrombocytopenia with a platelet count of 17 × 10^9^ cells/liter (reference rage, 125 × 10^12^ to 350 × 10^12^ cells/liter). His white blood cell count was 3.79 × 10^9^ cells/liter (reference rage, 3.5 × 10^9^ to 9.5 × 10^9^ cells/liter), differential with 81% neutrophilic granulocyte (reference rage, 40% to 75%) and 16% lymphocytes (reference rage, 20% to 50%). He was positive for HCV-antibody but HCV RNA was undetectable in his blood. Blood biochemistry showed an increased level of serum creatinine (501.5 *μ*mol/liter; reference rage, 40 to 160 *μ*mol/liter) and urea nitrogen (28.4 mmol/liter; reference rage, 2.2 to 8.3 mmol/liter) and a decreased level of albumin (25.4 grams/liter; reference range, 40 to 55 grams/liter). His CD4 cell count was 111 cells/microliter (reference range, 414 to 1123 cells/microliter) and HIV RNA level was 4.23 × 10^5^ copies/milliliter (reference range, <20 copies/milliliter).

Under the impression that he had severe septicemia, we initiated early goal-directed therapy and 4 separate blood specimens were sampled at 1-hour intervals consecutively for bacterial culture before empirical antibiotics were given. The patient was treated with 1 g cefoxitin every 6 hours and 10 mg dexamethasone every 12 hours intravenously, combined with blood transfusions and erythropoietin injections. The patient's condition stabilized the next day and his body temperature returned to normal 3 days later. The cefoxitin treatment was given for another 7 days and was discontinued when his white blood cell count returned to 2.51 × 10^9^ cells/liter (reference rage, 3.5 × 10^9^ to 9.5 × 10^9^ cells/liter) and two posttreatment blood cultures yielded negative results. Three weeks after this admission, his blood creatinine and urea nitrogen levels normalized, his anemia and thrombocytopenia improved, and he was discharged in a stable condition. No recurrence was reported on a follow-up 1 year after discharge.

All four blood cultures grew Gram-negative rods. In our laboratory, we used the MicroScan Walkaway (Siemens, Memphis, TN) for the identification of the organism and the results of a series of biochemical assays revealed it to be* Y. regensburgei* with 97.8% probability. It was positive for glucose, sorbitol, rhamnose, L-arabinose, melibiose, lysine, ornithine, citrate, galactoside, and cellobiose tests and negative for sucrose, raffinose, inositol, adonitol, urea, hydrogen sulfide, indole, arginine, tryptophan deaminase, aesculin, acetoin, malonate, tartrate, acetamide, cetrimide, and Voges-Proskauer tests. Susceptibility testing was performed using the MicroScan Walkaway and the Enterobacteriaceae criteria of CLSI were used by our laboratory [1]. The MIC results as shown in Table 1.

## 3. Systematic Review

We searched PubMed (https://www.pubmed.com/) with the keyword of* Yokenella regensburgei* on March 31, 2017, and found 10 articles. And then we used* Koserella trabulsii* as the keyword to search on PubMed on the same day and found another 6 articles. We reviewed all the 16 articles and found seven cases of* Y. regensburgei* infection as well as useful information about* Y. regensburgei*.

*Y. regensburgei* is one of a number of infrequent members of the family* Enterobacteriaceae* that have only rarely been isolated in humans. It was originally identified by Kosako et al. [2] through DNA hybridization in 1984 and later recognized by Hickman-Brenner et al. [3], working independently, in 1985, under the name of* Koserella trabulsii*. Subsequently, it was found that* Y. regensburgei* and* K. trabulsii* referred to the same* Enterobacteriaceae* and therefore the use of* K. trabulsii* has been dropped since 1991 [4].

*Y. regensburgei* closely resembles* Hafnia alvei* biochemically and has been misidentified as* Hafnia alvei* by automated systems [4]. It, therefore, has been hypothesized that infections due to* Y. regensburgei* have been underestimated due to misidentification of the bacterium. By studying susceptibility patterns and biochemical properties, Stock et al. [5] found that hydroxyproline amidase, maltosidase, tripeptidase, proline deaminase, catalase reaction, Voges-Proskauer test, and fermentation of glycerol, melibiose, and myo-inositol were suitable parameters to separate* Y. regensburgei* from* H. alvei*.* Y. regensburgei *is noted to possess amp C genes and express highly inducible, potent beta-lactamases and is intrinsically resistant to azithromycin and some beta-lactam antibiotics. However, it is weakly catalase positive and unable to produce hydroxyproline amidase, tripeptidase, or proline deaminase. Jachymek et al. [6] discovered novel trisaccharide repeating units of bacterial O antigens that are characteristic and unique to the* Y. regensburgei *species. Niedziela et al. [7] described the structures of the core oligosaccharides representing novel core types of bacterial LPS that are characteristic for* Y. regensburgei*.

*Y. regensburgei* appears to primarily belong to the bacterial flora of insects and has been recovered from the intestinal tracts of insects. Also, it has been isolated from the general environment such as in well water. Isolation of* Y. regensburgei* from a human specimen is rare and only seven cases of* Y. regensburgei* infection have been reported worldwide based on the literature we reviewed. Abbott and Janda [8] described two isolations of* Y. regensburgei* associated with extra-intestinal sites in humans immunocompromised due to alcohol abuse. The first isolate was from a left-knee wound of a 74-year-old male with a provisional diagnosis of a septic knee and a history of alcohol abuse. He was treated with amikacin, and no further data were available regarding the patient's clinical course. In the second case, a 35-year-old woman who abused alcohol suffered from an upper gastrointestinal bleeding. A blood culture grew* Y. regensburgei* during her hospital course, although she had no overt signs of sepsis such as fever or chills. The patent was subsequently treated with ciprofloxacin and released. Lo et al. [9] described a patient with membranous glomerulonephritis on immunosuppressive therapy with high-dose steroids (prednisolone total of 30 mg per day) and cyclophosphamide. The patient subsequently developed a soft tissue infection after abrasions to his leg, which had been contaminated with soil. He experienced fevers with chills, and his blood cultures grew* Y. regensburgei*. After receiving treatment with ceftriaxone for 3 weeks, he was discharged from hospital in stable conditions. Bhowmick and Weinstein [10] reported a 48-year-old male with multiple myeloma who had undergone an autologous stem cell transplant and had been on corticosteroids for liver disease. Later, the patient had a bulla on his right leg but had no fevers and chills. The bulla aspirate and 2 blood cultures grew* Y. regensburgei.* Fajardo Olivares et al. [11] described an 82-year-old male with chronic renal failure, venous thrombosis, and perimalleolar ulcer.* Y. regensburgei *was isolated from his ulcerous wound and he was cured with ciprofloxacin. Penagos et al. [12] presented a case of postsurgical secondary osteomyelitis due to* Y. regensburgei* in an immunocompetent woman who had undergone a craniotomy. The patient was successfully treated with ciprofloxacin for 42 days and there was no recurrence of infection at the end of 1-year follow-up. Jain et al. [13] reported a 5-year-old male child with continuous high-grade fever and chills for 7 days. Two blood cultures yielded positive results and the identification of both isolates was confirmed as* Y. regensburgei*. The child was treated with ciprofloxacin for 7 days and he responded clinically to the treatment. No recurrence was reported on a follow-up 3 months later.

## 4. Discussion

The patient in our present report, a confirmed HIV-infected individual with pancytopenia, was viremic in absence of ART when he was admitted due to a fever. The fact that* Y. regensburgei *was isolated from all 4 separate blood samples and the patient was successfully cured with cefoxitin demonstrated that the organism was definitely the cause of septicemia. Noticeably, the patient's white blood cell count was higher compared with his baseline values and the number decreased to its pretreatment levels after 10 days of antibiotic treatment, suggesting that* Y. regensburgei* infection could result in elevated white blood cell counts. We did not observe any evidence for the association between the patient's septicemia and his farming practice, although the organism could have been from the general environment like the soil. This is the first case of infection caused by* Y. regensburgei* in an HIV-infected patient to the best of our knowledge and our case supports the hypothesis that* Y. regensburgei *is an opportunistic pathogen in humans. However, we were not able to confirm the bacterial identification with sequencing, which is the limitation of this article. All the eight reported cases are summarized in Table 2.

Some common features can be observed from the seven reported cases and our case. Firstly, almost all cases have underlying diseases or immunocompromising conditions. Secondly, the majority of those cases have no systematic symptoms like fever and chills. Thirdly, the outcome of* Y. regensburgei* infection appears to be not too severe; only one death occurred among the eight cases, while five cases were cured. And lastly but interestingly, almost all the cases are from regions known for hot and humid weather.

In summary, we report a case of* Y. regensburgei* septicemia in a patient with HIV infection and pancytopenia. There are some similarities among the eight cases with regard to underlying conditions, clinical presentations, outcome, and geographical regions. From all the eight cases worldwide, it is reasonable to assume that* Y. regensburgei* is an opportunistic pathogen with a predilection to infect hosts severely immunocompromised by underlying diseases or conditions.

## Figures and Tables

**Table 1:** Susceptibility of the isolated *Y. regensburgei *stain.

Drugs	MIC	Susceptibility
Amikacin	≤8	S
Ampicillin	>16	R
Amoxicillin/clavulanate	≤8/4	I
Aztreonam	≤8	S
Ceftriaxone	≤8	S
Ceftazidime	≤1	S
Ceftazidime/clavulanate	≤0.25	S
Cefotaxime	≤2	S
Cefotaxime/clavulanate	≤0.05	S
Cefoxitin	≤8	S
Cefazolin	≤8	S
Ciprofloxacin	≤1	S
Cefepime	≤8	S
Piperacillin/tazobactam	≤16	S
Ertapenem	≤2	S
Gentamicin	≤4	S
Imipenem	≤1	S
Levofloxacin	≤2	S
Meropenem	≤1	S
Cefuroxime	≤4	S
Piperacillin	>64	R
Cotrimoxazole	≤2/38	S
Tetracycline	≤4	S
Ticarcillin/clavulanate	≤16	S
Tobramycin	≤4	S

S: susceptible; I: intermediate; R: resistance.

**Table 2:** Summary of *Y. regensburgei *infection cases reported worldwide.

Reference	Patient sex	Patient age (years)	Geographical region	Probable risk factor	Clinical specimen	Clinical diagnosis	Treatment	Outcome
Abbott and Janda (1994)	M	74	California, USA	Alcohol abuse	Left knee wound	Septic knee	Amikacin	Unknown

Abbott and Janda (1994)	F	35	California, USA	Alcohol abuse, liver disease, pancreatitis	Blood	Transient bacteraemia	Ciprofloxacin	Discharged, no follow-up

Fajardo Olivares et al. (2005)	M	82	Spain	Chronic renal failure, venous thrombosis	Wound	Perimalleolar ulcer	Ciprofloxacin	Cured

Lo et al. (2011)	M	42	Taiwan	Type 2 DM, renal disease, steroids, immunosuppressants	Blood	Cellulitis, sepsis	Ceftriaxone	Cured

Jain et al. (2013)	M	5	New Delhi, India	None	Blood	Enteric fever	Ciprofloxacin	Cured

Bhowmick and Weinstein (2013)	M	48	New Jersey, USA	Multiple myeloma, autologous stem cell transplant, corticosteroid use, liver disease	Blood, bulla aspirates	Soft tissue infection	Imipenem/cilastatin, clindamycin and gentamicin	Expired

Penagos et al. (2015)	F	70	Medellin, Colombia	Invasive pituitary macroadenoma, neurosurgery	Bone	Osteomyelitis	Ciprofloxacin	Cured

Index case	M	38	Chongqing, China	Acquired immunodeficiency syndrome, pancytopenia, drug abuse	Blood	Septicemia	Cefoxitin	Cured
